# Microbial Translocation and Gut Damage Are Associated With an Elevated Fast Score in Women Living With and Without HIV

**DOI:** 10.1093/ofid/ofae187

**Published:** 2024-03-30

**Authors:** Maria J Duarte, Phyllis C Tien, Ani Kardashian, Yifei Ma, Peter Hunt, Mark H Kuniholm, Adaora A Adimora, Margaret A Fischl, Audrey L French, Elizabeth Topper, Deborah Konkle-Parker, Howard Minkoff, Ighovwerha Ofotokun, Michael Plankey, Anjali Sharma, Jennifer C Price

**Affiliations:** Division of Gastroenterology and Hepatology, Department of Medicine, University of California San Francisco, San Francisco, California, USA; Department of Veterans Affairs Medical Center and Department of Medicine, University of California San Francisco, San Francisco, California, USA; Division of Gastroenterology and Liver Diseases, University of Southern California, Los Angeles, California, USA; Department of Veterans Affairs Medical Center and Department of Medicine, University of California San Francisco, San Francisco, California, USA; Division of Experimental Medicine, University of California San Francisco, San Francisco, California, USA; Department of Epidemiology and Biostatistics, University at Albany, State University of New York, Rensselaer, New York, USA; Department of Medicine, University of North Carolina at Chapel Hill, Chapel Hill, North Carolina, USA; Department of Medicine, Miller School of Medicine, University of Miami, Miami, Florida, USA; Department of Medicine, CORE Center/Stroger Hospital of Cook County, Chicago, Illinois, USA; Department of Epidemiology, Johns Hopkins Bloomberg School of Public Health, Baltimore, Maryland, USA; School of Nursing, Medicine and Population Health, University of Mississippi Medical Center, Jackson, Mississippi, USA; Department of Obstetrics and Gynecology, Downstate Health Sciences University, State University of New York, Brooklyn, New York, USA; Department of Medicine, School of Medicine, Emory University, Atlanta, Georgia, USA; Department of Medicine, Georgetown University Medical Center, Washington, DC, USA; Department of Medicine, Albert Einstein College of Medicine, Bronx, New York, USA; Division of Gastroenterology and Hepatology, Department of Medicine, University of California San Francisco, San Francisco, California, USA

**Keywords:** HIV-associated liver disease, MASLD, microbiome, steatohepatitis, steatotic liver disease

## Abstract

**Background:**

Steatohepatitis is common in persons living with HIV and may be associated with gut microbial translocation (MT). However, few studies have evaluated the gut-liver axis in persons living with HIV. In the Women's Interagency HIV Study, we examined the associations of HIV and circulating biomarkers linked to MT and gut damage using the FibroScan–aspartate aminotransferase (FAST) score, a noninvasive surrogate for steatohepatitis with advanced fibrosis.

**Methods:**

Among 883 women with HIV and 354 without HIV, we used multivariable regression to examine the associations of HIV and serum biomarkers linked to MT and gut damage (kynurenine and tryptophan ratio, intestinal fatty acid–binding protein, soluble CD14, and soluble CD163) with a log-transformed FAST score after adjusting for key covariates. We used a path analysis and mediation models to determine the mediating effect of each biomarker on the association of HIV with FAST.

**Results:**

HIV infection was associated with a 49% higher FAST score. MT biomarker levels were higher in women with HIV than women without HIV (*P* < .001 for each). MT biomarkers mediated 13% to 32% of the association of HIV and FAST score.

**Conclusions:**

Biomarkers linked to MT and gut damage are associated with a higher FAST score and mediate the association of HIV with a higher FAST score. Our findings suggest that MT may be an important mechanism by which HIV increases the risk of steatohepatitis with advanced fibrosis.

Metabolic dysfunction–associated steatotic liver disease (MASLD) is a major cause of liver-related morbidity and mortality, and its prevalence is enriched in people living with HIV (PLWH) [1–3]. While MASLD encompasses a range of histologic severity, metabolic dysfunction–associated steatohepatitis (MASH) has been associated with advanced fibrosis, cirrhosis, and hepatocellular carcinoma [4, 5]. Prior work evaluating liver biopsies in PLWH who did not have viral hepatitis coinfection reported a relatively high prevalence of nonalcoholic steatohepatitis (NASH) and significant liver fibrosis (≥F2), with pooled estimated prevalences of 42% and 22%, respectively, but lacked a comparison group of persons without HIV [6].

We previously found that among 1309 women without viral hepatitis (928 living with HIV and 381 without HIV), HIV infection was independently associated with a 3.7-fold higher odds of having an elevated FibroScan–aspartate aminotransferase (FAST) score, a noninvasive measurement of histologic MASH with an elevated nonalcoholic fatty liver disease activity score (≥4) and significant liver fibrosis (≥F2) [7]. Moreover, HIV viral suppression was associated with lower odds of an elevated FAST score [8]. These findings suggest that HIV increases the risk of MASH with advanced liver disease. Yet, the underlying mechanisms by which HIV influences the pathogenesis of MASLD and MASH remain unclear.

Recent studies have elucidated an important link between the gut microbiome and chronic liver disease, often termed the *gut-liver axis*. Translocated microbial by-products can activate hepatic Kupffer and stellate cells, leading to macrophage activation, inflammation, and fibrosis, with certain microbial signatures observed to be associated with MASLD in patients without HIV and in murine models [9–12]. HIV infection has been associated with profound dysbiosis and changes in gut permeability that persist despite antiretroviral therapy [4, 13–15], although gut microbial composition may be slightly modified by an antiretroviral therapy regimen [15]. PLWH have gut microbiomes with more pathogenic bacteria and less commensal bacteria when compared with HIV-seronegative controls [16]. HIV-associated dysbiosis has been linked to increased tryptophan catabolism, leading to an increase in catabolism by-products in the gut, most notably kynurenine. Kynurenine binds to T cells and inhibits differentiation of TH17 cells, thus leading to a reduction of interleukin 17 and 22 production. This in turn increases the permeability of the gut-mucosal barrier, allowing for potential translocation of microbes and their by-products [16].

Prior work has found an association between HIV and circulating biomarkers of microbial translocation, as well as links between microbial translocation and liver disease in people without HIV [17–20]. These studies support the hypothesis that microbial translocation and the resulting inflammatory response contribute to liver disease in PLWH. However, no studies to date have directly investigated how the gut-liver axis may be associated with the occurrence of MASH in PLWH.

## METHODS

### Study Population

Between 2013 and 2018, 1576 women living with and without HIV were enrolled in the Liver Disease and Reproductive Aging cohort, an ancillary study of the Women's Interagency HIV Study (WIHS; now part of the Multicenter AIDS Cohort Study [MACS]/WIHS Combined Cohort Study) [21]. Women without HIV (WWOH) who were demographically similar to women with HIV (WWH) and had risk factors for HIV within the past 5 years (ie, history of sexually transmitted infection, sex without a condom with ≥3 men, sex with a partner who was HIV seropositive, injection drug use) were recruited at the same time as women living with HIV. A unique strength of the WIHS cohort is its enrollment of demographically similar women who were HIV seronegative. Age, race, socioeconomic distribution, and behavioral habits at enrollment were similar to WWH in the cohort, who in turn were representative of US WWH [22].

The details of this cohort's and parent study's recruitment and inclusion/exclusion criteria have been described [21]. Briefly, all Liver Disease and Reproductive Aging participants underwent vibration-controlled transient elastography (VCTE; Echosens) and blood draw within 6 months of each other. VCTE-measured liver stiffness (kilopascals), controlled attenuation parameter (decibels per meter), and aspartate aminotransferase (international units per liter) were used to calculate the FAST score [7]. Women with positive hepatitis C virus antibody (anti-HCV) were excluded from the analysis, as were women with hepatitis B virus surface antigen. Each of the 13 participating institutions’ institutional review boards approved study protocols and consent forms, and all study participants gave written informed consent.

### Biomarker Testing

Plasma samples from specimens obtained within 6 months of VCTE measurement and stored at −70 °C were tested for the following biomarkers linked to microbial translocation and gut damage: kynurenine and tryptophan levels (to calculate a KT ratio, a marker of dysbiosis and microbial translocation), intestinal fatty acid–binding protein (I-FABP; a marker of gut epithelial integrity loss), and the immune activation markers soluble CD14 (sCD14; also a receptor for the bacterial by-product lipopolysaccharide) and soluble CD163 (sCD163; a protein produced by proteolytic cleavage of CD163 expressed on the surfaces of monocyte/macrophage lineage cells).

Kynurenine and tryptophan levels were measured with liquid chromatography–tandem mass spectrometry, as previously described [23]. I-FABP, sCD14, and sCD163 levels were measured by a commercially available enzyme-linked immunosorbent assay (Hycult Biotech and R&D), as previously described [24, 25].

### Covariates

Covariates were demographics (age [continuously], race/ethnicity), substance use behavior (current use of alcohol: none, light [1–7 drinks/wk], moderate [>7–12], or heavy [>12]; current use of drugs and tobacco; any history of injection drug use), and metabolic factors. These included body mass index (BMI; continuously), insulin resistance (continuously, calculated via the homeostatic model assessment for insulin resistance [HOMA-IR]), menopausal status (self-reported), and waist circumference (continuously; in centimeters). Among those with HIV infection, CD4 count (continuously), HIV viral load (detectable vs undetectable), CD4 nadir, history of AIDS, and current antiretroviral drug use were examined.

### Statistical Analysis

We performed chi-square and Student *t* tests to evaluate differences in demographics, substance use behavior, metabolic factors, and biomarkers of microbial translocation between WWH and WWOH.

FAST scores were log transformed to approximately conform to normality due to a right-skewed distribution, and we used multivariable linear regression to evaluate the association between HIV infection and log-transformed FAST score. We adjusted the model for covariates that were significantly different between WWH and WWOH in bivariate analysis, including age and menopausal status, as well as potential confounders that could influence the FAST score, including alcohol use, current tobacco use, race/ethnicity, BMI, and HOMA-IR. Regression coefficients were exponentiated to provide the percentage difference in FAST score between WWH and WWOH. After obtaining this baseline model, we added each microbial translocation biomarker to the model to determine its association with the FAST score. To compare the effect sizes across biomarkers linked to microbial translocation and gut damage with different units of measurement, we standardized units by log transforming each biomarker level and then dividing the log-transformed value by the log IQR.

Finally, we performed separate path and mediation analyses per biomarker to examine the percentage of the direct effect of the relationship between HIV and FAST score and the percentage of the mediating effect by each biomarker. We controlled for potential confounders in the mediation models: alcohol use, tobacco use, BMI, race/ethnicity, and menopause state. In addition, since the presence of HIV has been associated with insulin resistance in prior studies [26, 27], we added HOMA-IR to the mediation models to determine the percentage mediating effect of insulin resistance on the relationship between HIV and FAST score. The 95% CIs for mediating effects were obtained with a bias-corrected bootstrapping method. To account for missing data, we employed the full information maximum likelihood approach [28]. We used SAS version 9.4 and STATA version 17.0 to perform the analysis.

## RESULTS

### Demographics

After excluding women who did not have other known liver disease and were anti-HCV positive (n = 205), 1237 women (883 WWH and 354 WWOH) were included in a cross-sectional analysis. Overall, the median age was 49 years; most women (74.5%) were non-Hispanic Black; and over half were obese with a median BMI of 30.5 kg/m^2^ (Table 1). As compared with WWOH, WWH were more likely to have a greater median HOMA-IR and less likely to report being postmenopausal. In addition, WWH were more likely to report abstinence from alcohol than WWOH (52% vs 44%) and less likely to report heavy drinking (5.7% vs 12.7%, *P* < .001, across alcohol categories), smoking (37% vs 44%, *P* = .02), or a history of injection drug use (2.7% vs 5.7%, *P* = .01). Among WWH, the median CD4 count was 651 cells/mm^3^, and 91% reported undergoing antiretroviral therapy.

**Table 1.** Baseline Cohort Data by HIV Serostatus

	Median (IQR) or No. (%)			
	Entire Cohort (n = 1237)	Women Living With HIV (n = 883)	Women Living Without HIV (n = 354)	*P* Value^a^
Demographics				
Age, y	49 (43–54)	50 (44–55)	47 (41–54)	.008
Race/ethnicity				.197
Non-Hispanic White	116 (9)	92 (10)	24 (7)	
Non-Hispanic Black	921 (74.5)	653 (74)	268 (76)	
Hispanic	148 (12)	104 (12)	44 (12)	
Other	52 (4)	34 (4)	18 (5)	
Premenopausal	422 (34)	270 (31)	152 (43)	<.0001
Behaviors				
Current alcohol use				<.0001
Abstinence	614 (50)	458 (52)	156 (44)	
Light	484 (39)	345 (39)	141 (40)	
Moderate	42 (3)	30 (3)	12 (3)	
Heavy	95 (8)	50 (6)	45 (13)	
Current smoker	484 (39)	327 (37)	157 (44)	.017
Ever used injection drugs	44 (4)	24 (3)	20 (6)	.012
Metabolic				
Body mass index, kg/m^2^	30.5 (26–36)	30.4 (26–35.5)	31.3 (26.5–36.5)	.104
Waist circumference, cm	99.4 (89–111)	99.1 (89–111)	100.55 (88–113)	.71
HOMA-IR^b^	2.07 (1–4)	2.13 (1–4)	1.83 (1–3)	.032
Liver disease				
AST:ALT ratio	1.2 (1–1.44)	1.23 (1–1.5)	1.22 (1–1.5)	.410
Liver stiffness, kPA	4.9 (3.9–6.2)	5.1 (3.9–6.7)	5 (3.9–6.6)	.255
Steatosis: CAP score, dB/m	248 (207–281)	248 (211–291)	248 (210–289)	.399
Mild or greater steatosis, CAP ≥248 dB/m	691 (49)	485 (48)	206 (50)	.488
Significant fibrosis, ≥7.1 kPA	291 (23.5)	248 (24)	76 (18)	.105
Advanced fibrosis, ≥9.5 kPA	93 (7.5)	71 (8)	22 (6.2)	.120
Cirrhosis	24 (1.9)	22 (2.5)	2 (0.6)	<.001
HIV related	…		…	…
History of AIDS		265 (30)		
Currently taking antiretroviral therapy		802 (91)		
NNRTI		265 (30)		
Protease inhibitor		230 (26)		
INSTI		424 (48)		
HIV viral load, copies/mL		20 (20–32)		
Current CD4 count		651 (436–869)		
CD4 nadir		221 (104–360)		

Number of missing values: HOMA-IR, n = 203; waist circumference, n = 62; age, n = 2; body mass index, n = 2; CD4 nadir, n = 1.

Abbreviations: ALT, alanine aminotransferase; AST, aspartate aminotransferase; CAP, controlled attenuation parameter; HOMA-IR, homeostatic model assessment for insulin resistance; INSTI, integrase strand transfer inhibitor; NNRTI, nonnucleoside reverse transcriptase inhibitor.

^a^ *P* values express the difference between women living with HIV and without HIV. *P* values of continuous variables were calculated by unpaired *t* test. *P* values of categorical variables were calculated by a χ^2^ test.

^b^Insulin resistance is defined as HOMA-IR >2.0.

### HIV and Biomarkers Linked to Microbial Translocation and Gut Damage Are Associated With Higher FAST Score

On multivariable linear regression adjusting for age, BMI, race/ethnicity, HOMA-IR, menopause status, alcohol use, and tobacco use, HIV infection was associated with a 50% higher FAST score (95% CI, 29%–73.5%; Supplementary Table 1). Other factors associated with a higher FAST score were age (2% higher per year; 95% CI, 1%–3%), moderate alcohol use (53% higher vs no drinking; 95% CI, 6%–100%), and heavy alcohol use (53% higher vs no drinking; 95% CI, 15%–94%). Non-Hispanic Black race/ethnicity was associated with a 21% lower FAST score (95% CI, −37% to −.8%).

**Figure 1.**
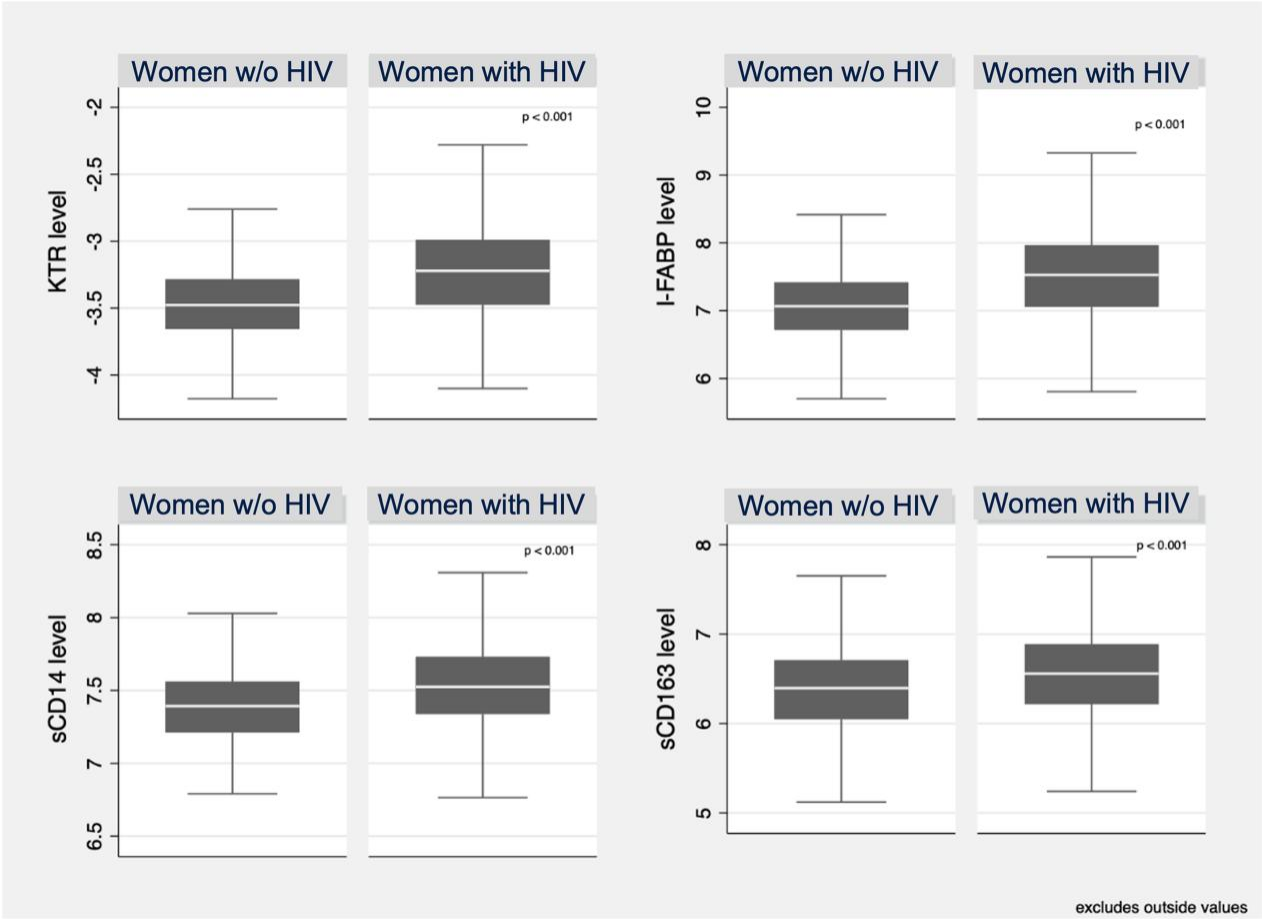
Levels of biomarkers linked to microbial translocation and gut damage by HIV serostatus. Log-transformed levels of plasma biomarkers are presented by HIV serostatus. Line, median; box, IQR; error bars, 95% CI. Outside values were excluded (n = 34) as follows: those below Q1 – 1.5 × IQR and those above Q3 + 1.5 × IQR. *P* values were calculated by *t* test. I-FABP, intestinal fatty acid binding protein; KTR, kynurenine-tryptophan ratio; sCD14, soluble CD14; sCD163, soluble CD163.

Levels of all biomarkers (KT ratio, I-FABP, sCD14, and sCD163) were significantly higher in WWH than in those without HIV (*P* < .001 for each biomarker; Figure 1). Specifically, median (IQR) biomarker levels were as follows: KT ratio, 3.86 (3–4.9) among WWH and 3.0 (2.5–3.6) among WWOH; I-FABP, 1843 pg/mL (1161–2866) and 1143 pg/mL (816–1639); sCD14, 1848 ng/mL (1537–2284) and 1613 ng/mL (1349–1897); and sCD163, 701 ng/mL (49–972) and 588.5 ng/mL (420–795; Supplementary Table 2). On multivariable linear regression, after adjusting for HIV status, age, BMI, race, HOMA-IR, alcohol use, tobacco, and menopause state, all biomarkers linked to microbial translocation and gut damage were significantly associated with a higher FAST score: the IQR increase of the log-scaled KT ratio was associated with a 17% higher FAST score (95% CI, 7%–28%), sCD163 with a 59% higher FAST score (95% CI, 46%–73%), I-FABP with a 12% higher FAST score (95% CI, 1.6%–23%), and sCD14 with a 17% higher FAST score (95% CI, 6%–28%; Table 2). This pattern was similar when stratified by HIV serostatus (Supplementary Table 3), and the difference between WWOH and WWH in the association between the biomarkers and FAST score did not show statistical difference (*P* = .78). After biomarkers linked to microbial translocation and gut damage were included as mediators, the association of HIV with increased FAST score was attenuated to varying degrees (Table 3).

**Table 2.** Association Between Standardized Plasma Biomarkers and FAST Score on Multivariable Analysis

	FAST Score, % Change per Biomarker IQR (95% CI)^a^	*P* Value
Kynurenine-tryptophan ratio	17.2 (7.4–27.8)	<.001
Intestinal fatty acid–binding protein	11.7 (1.6–22.9)	.02
Soluble CD14	16.6 (6.0–28.2)	.002
Soluble CD163	59.1 (45.9–73.4)	<.001

FAST score was log transformed. Biomarker levels were log transformed and underwent standardization, defined as the natural log-transformed variable divided by IQR.

Abbreviation: FAST, FibroScan–aspartate aminotransferase.

^a^FAST linear models were adjusted for HIV status, age, body mass index, race, insulin resistance, alcohol use, tobacco, and menopause state. Each biomarker was entered in the model separately.

**Table 3.** Direct Association of HIV and FAST Score With and Without Mediation Effect of Each Biomarker

	HIV Association, % (95% CI)
Fully adjusted model^a^	49.2 (28.7–72.9)
+ Kynurenine-tryptophan ratio	37.4 (17.9–60.2)
+ I-FABP	41.4 (21.2–64.9)
+ Soluble CD14	41.2 (21.5–64.1)
+ Soluble CD163	31.3 (13.8–51.6)

FAST score was log transformed and then exponentiated so that differences between regression coefficients on the log(e) scale can be expressed as proportions. The HIV effect (reference group: seronegative) on FAST score is reported via the baseline model (fully adjusted) and then with the addition of each biomarker to the model.

Abbreviations: FAST, FibroScan–aspartate aminotransferase; I-FABP, intestinal fatty acid–binding protein.

^a^Fully adjusted model includes body mass index, race, insulin resistance, alcohol use, tobacco, and menopause state.

### Microbial Translocation Mediates the Association of HIV on FAST Score

**Figure 2.**
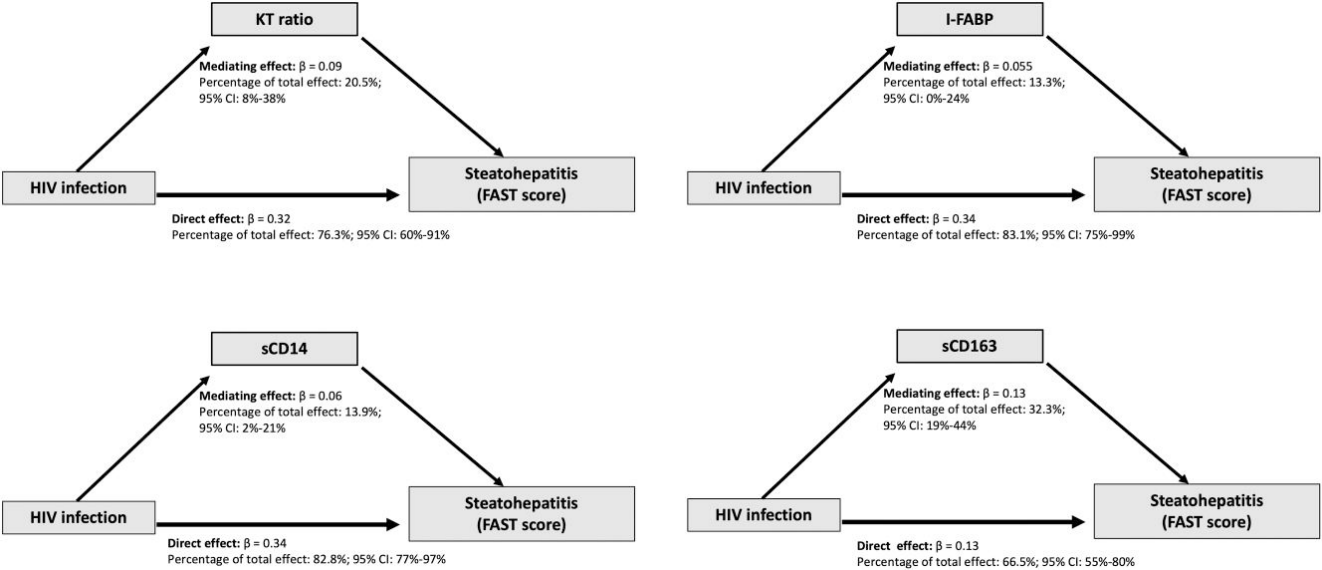
Path analysis of HIV, microbial translocation and gut damage, and FAST score. Path analysis shows multivariable adjusted effects of HIV infection status on steatohepatitis, as determined by FAST score. Standardized β coefficients for each mediator are shown, as well as percentages of the total effect attributable to microbial translocation and gut damage (microbial translocation as demonstrated by each biomarker) and non–microbial translocation pathways (other mechanisms of infection status, not including insulin resistance). The mediation effect of insulin resistance was also calculated by HOMA-IR and ranged from 1.3% to 3.6% (data not shown). Models are adjusted for HIV status, age, body mass index, race, insulin resistance (HOMA-IR), alcohol use, tobacco, and menopause state. The 95% CIs for mediating effects were obtained by a bias-corrected bootstrapping method. FAST, FibroScan-AST; HOMA-IR, homeostatic model assessment for insulin resistance; KT ratio, kynurenine and tryptophan ratio; I-FABP, intestinal fatty acid binding protein; sCD14, soluble CD14; sCD163, soluble CD163.

Since we found that increased microbial translocation biomarker levels were associated with HIV and higher FAST score and that there was attenuation of the HIV-FAST association with the addition of biomarkers to the models, we performed a path analysis to estimate the effect attributable to microbial translocation and insulin resistance in the relationship between HIV and FAST score. Bootstrap bias–corrected confidence intervals for each mediation effect revealed that the addition of 3 of the 4 biomarkers (KT ratio, sCD14, and sCD163) as mediators yielded a statistically significant attenuation in the association of HIV with FAST score. After adjusting for age, BMI, race, HOMA-IR, alcohol use, current tobacco smoking, and menopause state, the percentage of the HIV association with FAST score attributable to microbial translocation ranged from 13% (I-FABP) to 32% (sCD163; Figure 2). The total HIV effect on FAST score attributable to HOMA-IR ranged from 1.3% to 3.6% in each of the 4 path analysis models and was not statistically significant for any of the models.

## DISCUSSION

In this study of 1237 women with and without HIV, HIV infection was associated with higher circulating biomarkers linked to microbial translocation and gut damage. HIV infection was also independently associated with higher FAST score after adjustment for demographic, behavioral, and metabolic factors. A notable finding is that markers linked to microbial translocation and gut damage were strongly associated with higher FAST scores and mediated the association of HIV with higher FAST.

Our finding that HIV was associated with higher FAST scores is consistent with prior work from our group, which found significantly higher odds of elevated FAST score in WWH as compared with WWOH [8]. Our current study expands on the prior study by evaluating microbial translocation and gut damage as potential contributors to the higher prevalence of NASH in WWH. A noteworthy finding was the strong associations of all 4 biomarkers with higher FAST scores. This finding is consistent with mounting evidence that microbial translocation and MASLD are closely linked. In murine models, fecal microbiota transplant from mice with metabolic syndrome can induce metabolic syndrome and fatty liver disease in previously healthy mice [29], and human studies have shown that insulin function (which is strongly associated with MASLD) improved in participants who were obese after receiving a fecal microbiota transplant from donors who were lean [30].

Our study is consistent with established literature demonstrating a strong association between HIV infection and microbial translocation, as all biomarkers linked to microbial translocation and gut damage were higher in WWH [24, 31–33]. In addition, it is consistent with work demonstrating an association between liver fibrosis and microbial translocation in PLWH [17, 18]. In our cohort, we found an association of microbial translocation biomarkers with higher FAST score regardless of HIV serostatus, although I-FABP did not have a statistically significant association with FAST score in WWOH, possibly due to lower power. Overall, our findings are consistent with our hypothesis that microbial translocation may be a driver of MASLD-related inflammation in WWH. In addition, since WWH had higher levels of each biomarker, we hypothesized that microbial translocation may mediate the relationship between HIV and higher FAST score (Supplementary Figure 1). Indeed, the association of HIV with FAST score was attenuated after adjusting for each biomarker. Moreover, we observed that microbial translocation mediated up to 30% of the observed association of HIV with FAST.

Our findings were consistent regardless of the microbial translocation biomarker used, which is unsurprising given that each biomarker is representative of the same overall pathophysiology (translocation). It must be noted that several biomarkers linked to translocation and gut damage (KT ratio, sCD14, and sCD163) are also associated with immune activation in HIV. As such, they may be measuring multiple co-occurring pathways. It is therefore important to discuss the notable differences among the biomarkers. The tryptophan catabolism pathway (measured by KT ratio) and sCD163 affect immunoregulation and are associated with decreased T-reg differentiation, which may reflect a deeper level of injury to the liver from microbial translocation [34, 35]. Indeed, a prior study in WIHS found an association between higher KT ratio and increased liver fibrosis, as measured by FIB-4, among women living with HIV in the presence or absence of hepatitis C virus (HCV) infection [17]. In addition, sCD163 has been associated with indirect measures of liver fibrosis in WWH and HCV [36], as well as insulin resistance in PLWH [26]. In our study, sCD163 was associated with the highest indirect effect on FAST score of all the biomarkers. It may be that despite our adjustments for BMI and HOMA-IR, sCD163 may modulate the higher FAST score via microbial translocation and metabolic alterations, though notably our mediation analysis demonstrated an overall small indirect effect of insulin resistance (ranging from 1% to 3.6%) on the association between HIV and FAST score. This could point to alternate pathways that are mediated by sCD163, such as macrophage abundance, polarization, and activation and that affect MASH. In contrast, sCD14 is more directly tied with microbial translocation alone: it rises with macrophage activation and is the receptor for circulating lipopolysaccharide, a bacterial component [18, 19]. Elevated sCD14 has been associated with HCV-associated fibrosis in multiple studies in PLWH [18, 19, 37]. Notably, sCD14 is secreted from the liver and may be increased in liver disease independent from microbial translocation. The last biomarker that we evaluated, I-FABP, is a marker of gut turnover and can be a more specific marker of translocation as it is released with intestinal epithelial damage. I- FABP has been shown to correlate with progression of fibrosis to cirrhosis in HIV/HCV coinfection [18, 31].

There are important limitations to our study. Given the cross-sectional design, it is not possible to completely elucidate a causal path by which microbial translocation may mediate the effect of HIV on higher FAST score. Furthermore, although prior literature has established each biomarker as being strongly correlated with microbial translocation, the complex physiology and immunoregulation of each marker make it challenging to state with certainty that they are each representative of only microbial translocation, and there may be unknown or unmeasured confounders. The use of 4 separate proxies for translocation does allow us greater confidence in our conclusions, as they performed similarly despite being evaluated independently. In addition, while the FAST score has been validated in large cohorts excluding PLWH, it has not been validated as a surrogate for histologic MASH in PLWH. However, an elevated FAST score predicts liver-related outcomes among PLWH [38]. In addition, persistent inflammation in PLWH may contribute to liver and multiorgan fibrosis and may be driving elevated FAST scores. While microbial translocation may play a role in this persistent inflammation, further studies are necessary to better elucidate this. Furthermore, WWH had lower alcohol consumption than WWOH in our study population. Since alcohol use can increase the FAST score in the absence of MASH, some WWOH may have had falsely elevated FAST scores. However, we controlled for this by adjusting for alcohol consumption in the base model. In addition to this, a limitation of our mediation analysis is that it relies on several key assumptions and control of confounders. While we adjusted mediation models for known confounders, there may be unmeasured confounding that is not being taken into account. Finally, our study may not be generalizable to men living with HIV: there are data demonstrating that sex and menopause state influence the natural history of chronic liver disease, including hepatitis C and MASLD [39, 40].

In conclusion, our findings suggest that microbial translocation may be an important mechanism by which HIV increases the risk of MASH with advanced fibrosis. Prospective studies and further microbiome analyses, including stool genomic sequencing, will be critical to explore this potential pathway. Ultimately, the clinical implications of these findings and future studies could include manipulation of the microbiome to help improve MASLD outcomes.

## Supplementary Material

ofae187_Supplementary_Data

## Supplementary Data

Supplementary materials are available at *Open Forum Infectious Diseases* online. Consisting of data provided by the authors to benefit the reader, the posted materials are not copyedited and are the sole responsibility of the authors, so questions or comments should be addressed to the corresponding author.

## Notes

***Acknowledgments.*** Data in this article were collected by the Women’s Interagency HIV Study, now the MACS/WIHS Combined Cohort Study (MWCCS). The authors gratefully acknowledge the contributions of the study participants and dedication of the staff at the MWCCS sites.

***Author contributions.*** All authors made substantial contributions to conception and design, acquisition of data, or analysis and interpretation of data, as well as drafting the article or revising it critically for important intellectual content, and all gave final approval of the version to be published.

***Disclaimer.*** The contents of this publication are solely the responsibility of the authors and do not represent the official views of the National Institutes of Health.

***Patient consent statement.*** All study participants gave written informed consent, and each of the 13 participating institutions’ institutional review boards approved study protocols and consent forms: University of California San Francisco, University of Southern California, University at Albany, State University of New York, University of North Carolina at Chapel Hill, University of Miami Miller School of Medicine, Stroger Hospital of Cook County, Johns Hopkins University, University of Mississippi, State University of New York Downstate Health Sciences University, Emory University School of Medicine, Georgetown University Medical Center, and Albert Einstein College of Medicine.

***Previous presentation of material***. Preliminary data from this study were presented by M. J. D. at the 2023 Digestive Disease Week Conference, Chicago, Illinois, 7 May 2023.

***Financial support***. This work was supported by the National Institutes of Health (T32DK060414 to M. J. D.), with support to each MWCCS clinical research site as follows (author/principal investigator): Atlanta (I. O.; U01-HL146241), Baltimore (U01-HL146201), Bronx (A. S.; U01-HL146204), Brooklyn (U01-HL146202), Data Analysis and Coordination Center (E. T.; U01-HL146193), Chicago–Cook County (A. L. F.; U01-HL146245), Chicago-Northwestern (U01-HL146240), Northern California (J. C. P. and P. T.; U01-HL146242), Los Angeles (U01-HL146333), Metropolitan Washington (U01-HL146205), Miami (M. A. F.; U01-HL146203), Pittsburgh (U01-HL146208), University of Alabama at Birmingham (D. K.-P.; U01-HL146192), and University of North Carolina (A. A. A.; U01-HL146194).

The MWCCS is funded primarily by the National Heart, Lung, and Blood Institute, with additional cofunding from the Eunice Kennedy Shriver National Institute of Child Health and Human Development, National Institute on Aging, National Institute of Dental and Craniofacial Research, National Institute of Allergy and Infectious Diseases, National Institute of Neurological Disorders and Stroke, National Institute of Mental Health, National Institute on Drug Abuse, National Institute of Nursing Research, National Cancer Institute, National Institute on Alcohol Abuse and Alcoholism, National Institute on Deafness and Other Communication Disorders, National Institute of Diabetes and Digestive and Kidney Diseases, and National Institute on Minority Health and Health Disparities, in coordination and alignment with the research priorities of the National Institutes of Health’s Office of AIDS Research.

MWCCS data collection is also supported by UL1-TR000004 (UCSF CTSA), UL1-TR003098 (JHU ICTR), UL1-TR001881 (UCLA CTSI), P30-AI-050409 (Atlanta CFAR), P30-AI-073961 (Miami CFAR), P30-AI-050410 (UNC CFAR), P30-AI-027767 (UAB CFAR), P30-MH-116867 (Miami CHARM), UL1-TR001409 (DC CTSA), KL2-TR001432 (DC CTSA), and TL1-TR001431 (DC CTSA).
