# Supplementary material for: Microbial Translocation and Gut Damage Are Associated With an Elevated Fast Score in Women Living With and Without HIV
Source: Open Forum Infect Dis. 2024 Mar 30;11(5):ofae187. doi: 10.1093/ofid/ofae187 (PMC11055391; doi:10.1093/ofid/ofae187)
Supplement: ofae187_Supplementary_Data [file ofae187_supplementary_data.zip › FAST_MT_Supp_Table1.docx]

**Supplemental Table 1: Multivariable analysis of FAST score, baseline Model (Entire study population)**

| **Covariate** | Percent change in FAST score | 95% CI | p-value |
| --- | --- | --- | --- |
| **HIV** | 49.2 | 28.7 - 73 | <0.001 |
| **Age (per year)** | 2.1 | 1 – 3.2 | < 0.001 |
| **BMI (per kg/m^2^)** | 0.8 | -0.1 – 1.7 | 0.096 |
| **Black** | -22.1 | -38.1 - -2 | 0.03 |
| **Hispanic** | -19.9 | -39.8 – 6.6 | 0.13 |
| **Other Race** | -7.9 | 37.4 – 35.5 | 0.67 |
| **HOMA-IR (per doubling)** | 8.9 | -0.3 – 19 | 0.06 |
| **Alcohol** |  |  |  |
| **Light** | 9.6 | -4.9 – 26.2 | 0.2 |
| **Moderate** | 52.6 | 5.8 – 100 | 0.02 |
| **Heavy** | 52.5 | 17 – 99 | 0.0019 |
| **Current Tobacco Use** | -2.5 | -15 – 12 | 0.72 |
| **Menopause** |  |  |  |
| **Post- Menopause** | -1.7 | -19 – 19.3 | 0.86 |

**Abbreviations**: CI, 95% confidence interval, FAST, FibroScan- aspartate aminotransferase Score; Homeostatic Model Assessment for Insulin Resistance

All covariates listed in the table were included in the model

To account for missing data, we employed the full information maximum likelihood (FIML) approach. Number of missing values: HOMA-IR 203; Age 2; BMI 2

FAST and HOMA-IR scores were log transformed
