# Supplementary material for: Microbial Translocation and Gut Damage Are Associated With an Elevated Fast Score in Women Living With and Without HIV
Source: Open Forum Infect Dis. 2024 Mar 30;11(5):ofae187. doi: 10.1093/ofid/ofae187 (PMC11055391; doi:10.1093/ofid/ofae187)
Supplement: ofae187_Supplementary_Data [file ofae187_supplementary_data.zip › FAST_MT_Supp_Table3.docx]

**Supplemental Table 3: Association between standardized serum biomarkers and FAST score on multivariable analysis by HIV serostatus**

|  | **FAST score (% change per biomarker IQR, 95%CI)**** | | |
| --- | --- | --- | --- |
|  | **Women with HIV** | **Women without HIV** | |
| **KT ratio** | 12.4% (1%, 25%)* | | 22% (0.3%,48.5%)* |
| **I-FABP** | 14.2% (2.5%, 27%)* | | 15.1% (-6%, 41%) |
| **sCD14** | 12.3% (0.5%,25%)* | | 25.1% (3.3%, 51%)* |
| **sCD163** | 58.1% (42%,75%)* | | 58.6% (34%, 88%)* |

**Abbreviations**: CI, 95% confidence interval, FAST, FibroScan- aspartate aminotransferase Score; KT, Kynurenine -Tryptophan; I-FABP, intestinal fatty acid binding protein; sCD14,soluble CD14; sCD163,soluble CD163.

FAST score was log-transformed. Biomarker levels were log transformed and underwent standardization defined as the log transformed variable divided by IQR

**FAST linear models were adjusted for HIV status, age, BMI, race, insulin resistance (HOMA-IR), alcohol use, tobacco, and menopause state. Amongst WWH the models were also adjusted for CD4 count, HIV viral load and protease inhibitor use

Each biomarker was entered in the model separately

No significant interaction was observed between HIV serostatus, biomarker levels and FAST score

*p<0.05
