# Supplementary material for: Microbial Translocation and Gut Damage Are Associated With an Elevated Fast Score in Women Living With and Without HIV
Source: Open Forum Infect Dis. 2024 Mar 30;11(5):ofae187. doi: 10.1093/ofid/ofae187 (PMC11055391; doi:10.1093/ofid/ofae187)
Supplement: ofae187_Supplementary_Data [file ofae187_supplementary_data.zip › FAST_MT_Table2.docx]

**Table 2: Association between standardized plasma biomarkers and FAST score on multivariable analysis**

|  | **FAST score (% change per biomarker IQR, 95%CI)*** | **p-value** |
| --- | --- | --- |
| **KT ratio** | 17.2% (7.4%, 27.8%) | <0.001 |
| **I-FABP** | 11.7% (1.6%, 22.9%) | 0.02 |
| **sCD14** | 16.6% (6.0%, 28.2%) | 0.002 |
| **sCD163** | 59.1% (45.9%, 73.4%) | <0.001 |

**Abbreviations**: CI, 95% confidence interval, FAST, FibroScan- aspartate aminotransferase Score; KT, Kynurenine -Tryptophan; I-FABP, intestinal fatty acid binding protein; sCD14,soluble CD14; sCD163,soluble CD163.

FAST score was log-transformed. Biomarker levels were log transformed and underwent standardization defined as the natural log transformed variable divided by IQR

*FAST linear models were adjusted for HIV status, age, BMI, race, insulin resistance (HOMA-IR), alcohol use, tobacco, and menopause state. Each biomarker was entered in the model separately.
