# Supplementary material for: Microbial Translocation and Gut Damage Are Associated With an Elevated Fast Score in Women Living With and Without HIV
Source: Open Forum Infect Dis. 2024 Mar 30;11(5):ofae187. doi: 10.1093/ofid/ofae187 (PMC11055391; doi:10.1093/ofid/ofae187)
Supplement: ofae187_Supplementary_Data [file ofae187_supplementary_data.zip › Supplementary Figure 1.docx]

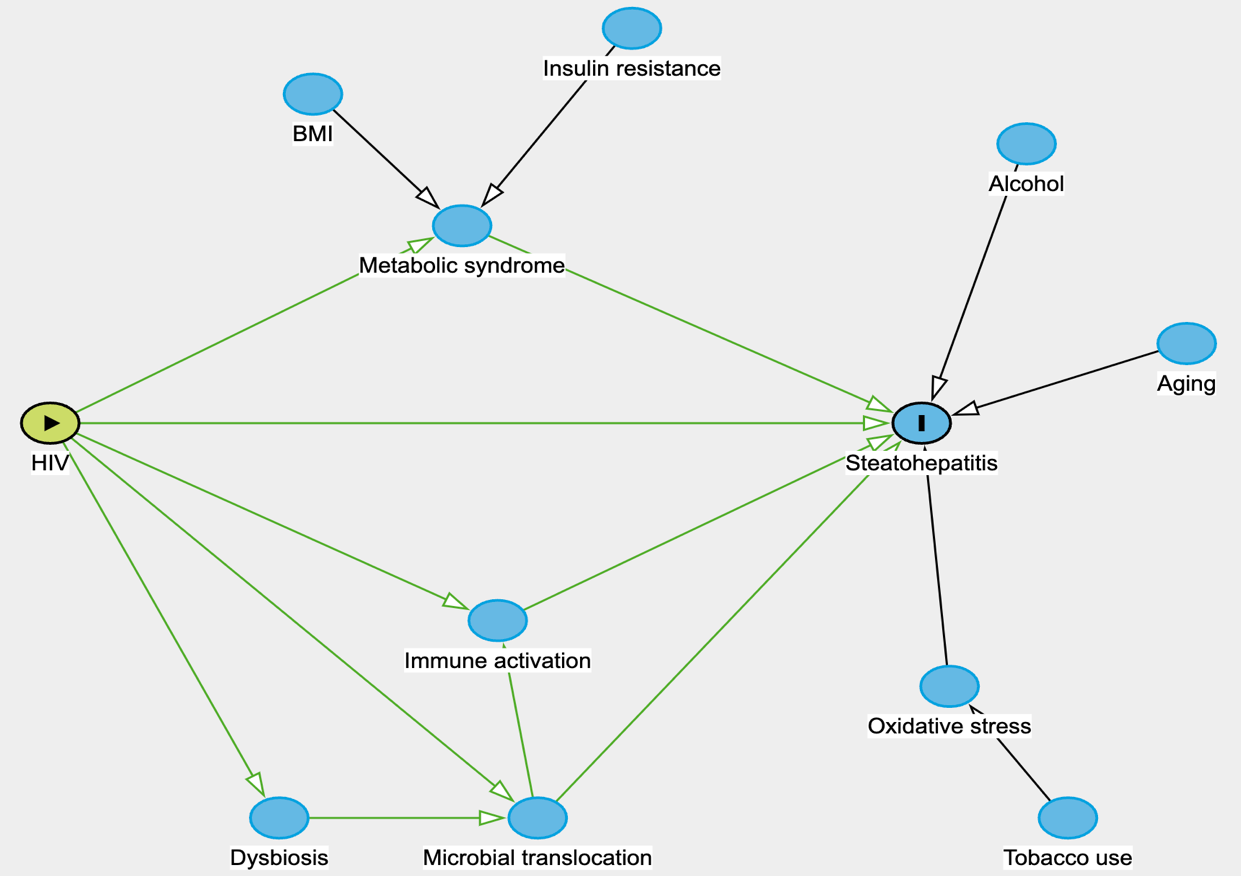


**Supplementary Figure 1:** Directed acyclic diagram (DAG) demonstrating the relationships between chronic HIV infection (in green), microbial translocation and steatohepatitis. Green arrows represent major pathways in this relationship, and black arrows represent other relevant pathways which are controlled for in the models.
