# Supplementary figures and images for: Microbial Translocation and Gut Damage Are Associated With an Elevated Fast Score in Women Living With and Without HIV
Source: Open Forum Infect Dis. 2024 Mar 30;11(5):ofae187. doi: 10.1093/ofid/ofae187 (PMC11055391; doi:10.1093/ofid/ofae187)

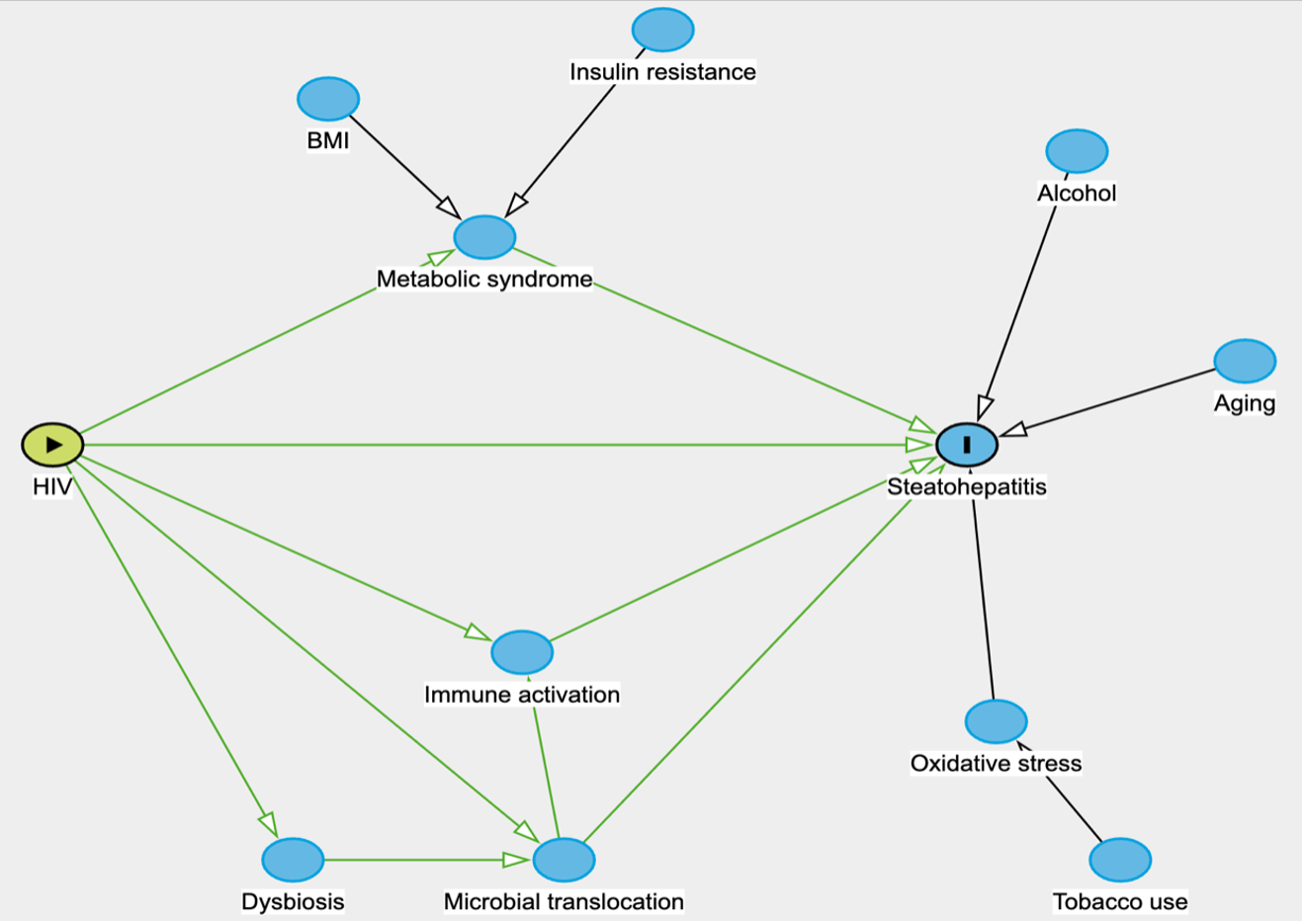

Supplement: ofae187_Supplementary_Data [file ofae187_supplementary_data.zip › Supplementary_Figure_1_TIFF.tiff]
